# Dentistry and Preventive Hygiene

**Published:** 1909-04-15

**Authors:** Horace Fletcher

**Affiliations:** New York and Venice, Italy


### DENTISTRY AND PREVENTIVE HYGIENE.

BY MR. HORACE FLETCHER, NEW YORK AND VENICE, ITALY.

This liberal abstract is taken from an address published in the February *Cosmos*, which was read at Rochester, N. Y., November 14, 1908, before the 7th and 8th District Dental Societies.—Editor REGISTER.

I want to give you my most optimistic idea of what dentistry is best fitted to be, and what I think it will be considered within the next ten years. I believe that dentistry within the next ten years will be looked upon as the special department of the hygienic professions whose office will be the conservation of the front gateway of the alimentary canal in more ways than the mere care of the teeth. I believe that medicine, in relation to dietetics, will have become a curiosity of history, and that the human equipment for nourishment will be put under two special departments, the mental and the dental both under the care of the dentist.

Some years ago I wrote an essay upon the combining of the two specialties of Medicine and Divinity. I prophesied that these two callings would eventually be merged into one; that it was quite necessary for the spiritual adviser to also be an adviser in hygiene, and quite as necessary that the doctor of hygiene should have some idea of the spiritual yearnings of the individual being treated. And this has come about—not as the result of my essay, because I did not publish it; still, at the same time, while I was writing the essay I was disseminating these ideas by wireless, and anyhow they were "in the air," and perhaps my note helped the suggestion to materialize.

Sometime, when I go to Boston, if I am happy enough to see Dr. Worcester, I am going to ask him some questions, and I expect he will reply to my questions in a manner satisfactory to the dental profession. I hope this reply will be, "When people come to me for mental consultation the first question I ask is, Have you a good set of teeth? If the teeth are not in a perfectly satisfactory condition, I want to know how bad they are, and I direct them to have an examination to see if there is any evidence of Riggs' disease, or if exuding pus is being swallowed constantly or not. I will make these inquiries because the tooth question and the question of therapeutics, either physical or metaphysical, are very closely connected." If he finds an imperfect dental equipment, he will advise the patient to go to a dentist for treatment if he is able to pay, or to one of the free dental infirmaries if he be too poor to employ a dentist.

The relation of the mental to the dental is this: You may create all the favorable suggestion you like, but unless you have a good set of teeth with which to masticate your food properly, in order to allow the juices of the mouth to get at it, or unless the teeth are in condition to munch your food comfortably and enjoyably, you are sure to have poor digestion. This has been shown conclusively by Pavloff and Cannon in experiments with which you are all probably familiar.

I am not going to give you a long dissertation on the method which I advise in taking food, because it has already been published so widely that you cannot have escaped learning about it. All of our study of the question leads in the direction, not of extending, but of simplifying our personal responsibility in the matter of our own nutrition. In considering a way by which simplification can be aided, Dr. Van Someran of Venice has suggested that we can profitably divide digestion into two departments, the *voluntary* and the *involuntary*. Voluntary digestion is that which takes place while the food is yet under control and before it is swallowed. The results of our experiments for the past ten or twelve years have shown that when food is properly treated within the small section of the alimentary canal under our voluntary control, we have no evidence apparent to the senses, that there is any more alimentary canal beyond the throat. We forget that we have a stomach, forget that we have intestines, and the whole process of involuntary digestion is done so completely and easily that we have no thought or care in the matter at all. What we want to do is to concentrate our attention on the things which are our own particular responsibility and let nature do her self-assigned part of the work uninterrupted, uninterfered with, and unquestioned.

Consider the complexity of that small three inches of the alimentary canal and how much of importance happens in and around the mouth! There is where nearly all the sensations are expressed. Nature does everything she can do to concentrate your attention there, where is enjoyed all the pleasure of eating and where she first protests if the act of eating has been careless. If there be trouble in the field of involuntary digestion, in the stomach or other intestines, it is in the mouth that the acids and ferments of indigestion, are first observed. In the mouth we sense all the pleasure of eating; there is where is concentrated our consideration of true appetite, "watering of the mouth," and there is where nature has done everything to attract our attention; but man, with the perversity of a perverse child, has disregarded this evident advice, these beneficent precautions and allurements. We have ignored these attempts to concentrate attention upon this portion of the canal, and we have been groping for remedies to counteract the results of our carelessness in the dark recesses of the involuntary field. The medical profession has been directing our attention elsewhere, so that we have been roaming about in uncertainty and confusion over the thirty or more feet of the alimentary canal where we do nothing but mischief, and have utterly neglected the three inches which are our field of personal responsibility.

When I took up the study of this subject ten or twelve years ago—that is, concentrating my own independent thought on it—I went first to the books for information. I found, even at that time, scores of treatises upon dietetics, every one of which was written, not to proclaim an undoubted truth, but to deny some other theory, and no two were in concurrence. Then I turned away from the field of dietetic speculation and opened anew my physiological text-book to review my study of the subject of years ago, and I found only a few pages devoted to the three inches of the alimentary canal, which is our personal responsibility, but nearly three thousand pages devoted to what is supposed to happen after food is swallowed, beyond the field of our responsibility. It occurred to me, as a person who had had some experience with business, as a person having been taught to apply strict methods of analysis to business problems, "Is it possible that this important section of our anatomy, which is practically the protective gateway of our nutrition, the laboratory of our efficiency, has been carelessly overlooked in regard to its basic importance? Is it true that the scientific world has neglected this important tract so completely and has devoted so much time to working in the field which nature has reserved for her own responsibility?" I went over the literature of the subject and found that nobody had given an undoubtedly logical solution. So I took up the study of the subject myself, from a purely business point of view, and began to investigate the anatomy of the mouth and what takes place there. An account of my discoveries may be found in my earlier books.*

Food is taken into the mouth and the process of mastication and insalivation begins. The tongue moves about and pushes the food between the teeth and up against the roof of the mouth. Mixture of saliva with food causes a chemical transformation which gives us taste. As insalivation progresses the food becomes alkaline, or neutral, or whatever is necessary to make it acceptable to the body. There is a furrow in the center of the tongue, and when the food becomes liquified it crawls up the furrow until it comes to the gate of the throat in the region of the circumvallate papillae. If it is in a condition acceptable to the discriminating sense, which I believe is associated intimately with the circumvallate papillae, the closure is relaxed, the soft palate hanging down behind the thick part of the tongue acts like the sucker of a pump, and draws the chemically transformed food material back for swallowing.

Suppose you take your meal (a piece of dry bread, for instance) with your head down; when you begin the process of mastication you can feel the saliva mixing with the bread until finally it becomes very creamy and sweet. When it attains that state, you will note that it begins to crawl up the concavity of the tongue, "runs up-hill," as it were, against gravitation, and when it arrives at the vicinity of the circumvallate papillae there is a slight sensation of contact, an inclination to open up the gate. This is the important discriminating apparatus and function which I claim to have discovered, and which I have named "Nature's food filter." Unless forcibly prevented by the will, the gate then opens, and the food is drawn back by the negative pressure behind it (suction), and the reflex of deglutition is set

*"The New Glutton or Epicure." The Frederick A. Stokes Company, New York, N. Y.

up and completed. At this moment the larynx is brought forward under the base of the tongue for the protection of the air-passages, the pharynx is brought into convenient place, and peristalsis follows.

My own inference in the matter is that, while there is any taste left in a morsel of food in the mouth the closure remains, but with the disappearance of taste there is a report sent to the brain by the nerves surrounding the circumvallate papillae that the chemical transformation is completed; the message is sent to the brain through the nerves connected with the muscles, and a message is sent back for the gate to open, and the process of deglutition is begun. This is the process called "involuntary swallowing."

Now, I want to tell you that ten or twelve years of careful attention to observation of this discriminating process and to the avoidance of swallowing anything that does not *swallow itself*, using the filtering apparatus as nature intended, has resulted in the disappearance of all disabilities of digestion. If anyone will for a week or ten days carefully use the filter-function in the way I have recommended, eating only food which appeals to the appetite, faithfully masticating or sipping it; swallowing nothing except by the involuntary process; ceasing to eat when no longer actually hungry; discarding anything that is not swallowed involuntarily, the filter-function will be sensitized so perfectly that it can be trusted to work automatically. But at this point do not think that your responsibility has ceased. Whereas chewing and care are important, the one thing of greatest importance is the mental attitude toward the food you are eating and your mental state during the time the food is being digested.

Those of you who have followed Dr. Cannon's experiments during the past ten years have learned from his report of them that the mental state may either accelerate the process of digestion in the alimentary canal, retard it, or even stop it entirely. To illustrate this I will give you a brief description of some of Dr. Cannon's experiments, which no doubt will be interesting in this connection. He uses cats as test subjects. The alimentary canal of the cat is luminous to the X ray throughout. The food is mixed with subnitrate of bismuth to render it opaque to the X ray. Professor Cannon allows his cats to become very hungry, with the appetite unusually strong. Then he selects the food his cats seem to like. Before giving it to a cat the food is covered with subnitrate of bismuth, and consequently when it passes into the stomach through the gullet, the shadow from the X ray tells what is happening on the way. I have watched the process, and it has been pictured by the photographic method. The cat, in not having much on its mind except the desire for something to eat, is fullfilling all the demands of nature that are required. There is first of all a strong appetite, and when the cat takes food, he is pleased with the enjoyment of it, and is thus fulfilling the psychic requirements of his nature. As he swallowed his food we watched the shadow as it went down the esophagus, hastened by peristalsis, and all this time the cat was lying on the fluorescent screen, contended and happy, as if before a log-wood fire. He was in a fine frame of mind and began to purr. During the purring the food was on its way into and through the stomach. Finally, as the food came to the pyloric orifice (the back-gate of the stomach) it began to hesitate a moment, then all at once we saw a small portion go through the opening, while the rest continued moving about and around the orifice. Then more of the food went through, and the process continued until all had left the stomach.

When it came to the duodenum we commenced to see the process of assimilation pctured by the shadow. The papillae conniventae are seen to reach down and pick up the digested particles of food, and this goes on at something like the rate of five hundred dips a minute while the cat is still purring and happy. Then, for the purpose of experiment and not through mischief, the cats' attention is distracted, and at once the movement slows up. The cat is further irritated, and immediately the whole process ceases; everything becomes rigid. It is paralysis of digestion due to mental influence. If we restore the amiability of the cat —if it is made happy and the purring begins again—mind you, the process of digestion does not immediately resume; it takes considerable time before ordinary digestion commences again. Moreover, when the digestive process is resumed, it is less active than that which takes place before the irritation is imposed. What is the result of this? The moment the digestive juices cease to flow, the bacteria of putridity in the alimentary canal are given a chance to do their deadly work. They begin at once, and that means trouble. They turn the inert food material, not into nutriment, but into poisons; the poisonous material is taken up by the blood and the lymph streams.

Now I will tell you how this illustration, taken from Dr. Cannon's researches, relates to ourselves. The same laws of nature which govern the digestive process in the cat affect us. If we take food in a hasty manner, without due appreciation of appetite and enjoyment, with our thoughts on business or on the catching of a train, and more particularly if we are irritated to the extent of having a "scrap" at the breakfast table, indulging in discussions about meals, business, politics, or anything whatever, we may be sure that during the time we are so doing we are manufacturing poison which is being sent through the body. I think I may illustrate the importance of the psychological influence on digestion by an experiment of Professor Pavloff. He was able through his skill as a surgeon to sever the gullet of his dog test-subject so that the mouth was separated from the rest of the alimentary canal, and to the end of the severed gullet a rubber tube was attached. The dog was allowed to get very hungry. He was then given food, and ate it as a dog will do, but instead of the food going into the stomach it went through the rubber tube and back into the dish. In this way the dog kept on eating without satisfying in any measure his appetite. The moment he sensed enjoyment of the food being eaten a copious flow of the juices in the stomach was started, in anticipation of the food arriving in the stomach. This anticipatory flow went on the whole time the dog was eating and enjoying the taste of his food. An interesting feature of this experiment is that it shows us how necessary is the enjoyment of food in the mouth to secretion of gastric juice in the stomach. If food be forced into the stomach of a dog directly through the lower end of the severed tube, without the enjoyment of eating, there is very little or no response from the source of gastric supply.

I have told you something of what happens in the mouth. If you get full enjoyment of your food through tasting it thoroughly, digestion will be perfect; and what is the result? This is very important. If you take your food in the manner I recommend—if you swallow only by involuntary swallowing (and it is only by these means that you can get keen enjoyment of your food); if you then stop when appetite slackens—you will find that you no longer are being reminded, through discomfort, that you have anything in the form of a stomach, and all the abnormal cravings which are mistaken for hunger will disappear entirely. These uncomfortable stomach sensations are merely pathological conditions in connection with indigestion, and are really in themselves forms of indigestion. Real hunger has nothing to do with "faintness" or "all-goneness." If you take food in the manner intended by nature, and as I recommend, you will find that appetite is a perfect guide; it is the true language of the body. You may not know what proteid or starch is, or what food contains mineral salts, but appetite will lead you to the proper selection from almost any available supply. The language of nature is not the language of calories, nor is it expressed in terms of proteids.

Having learned this lesson, having taken food in the manner recommended, having satisfied normal appetite, what will be the result? You will find that health is secure and energy abundant. And why? You will have ceased to produce poisons in the body. You will find that one-half or one-third of the amount of food you have been accustomed to taking will better satisfy your appetite and will nourish you better—so much so that even thin people who are under weight will begin to gain weight on perhaps half the food they have been taking. And what is the reason? The reason is, that the body under this care utilizes everything accepted by it; expending energy on superfluous material will cease, and consequently it will be available for work or play. It is like finding pure water after having suffered from using water from polluted sources. When this is done, what is the result? The thing of greatest importance, which can only be observed and understood by the individual himself, is that there is now thorough digestion of food, right nutrition of the body, no putrid decomposition, no poisonous products, no offensive odors. The normal process in man, woman, or child is a cleanly process, a process which has nothing of offensiveness about it. Take the excretions of the body (perspiration, for instance, which in some instances is very offensive), they will become no more offensive than those of a babe. And why? Simply because the products being excreted are not putrid, but merely the natural waste of healthy metabolism. I do not know anything more satisfactory than having it within one's own power to be cleanly inside as well as outside, and if cleanliness inside is first attended to, outward cleanliness is a matter of course.

I have given you but an inadequate idea of the normal process of nutrition, but it reduces itself to this simple knowledge and formula: Never eat until you are hungry; never eat when in a disturbed state of mind; never allow worry, anger, or fear to possess you during the time you are serving on the holy altar of your nutrition—the source of your efficiency. It is clearly a sacred process; it means physical and moral cleanliness; it is most sacred of duties. When you have done all that you can do to safeguard your nutrition, you may assure yourself that you will be always in the "pink of condition," whether you are an athlete in faithful training or not. You will find your energy enhanced, and your muscle endurance will increase from fifty to two hundred per cent. according to the room for improvement. You will cease to have disagreeable symptoms of headache or lack of energy. Fatigue will practically become a thing of the past. Sleepiness will be the only symptom of fatigue. You will find that your muscles have improved, through having been given pure nourishment, to the extent, may be, of two hundred per cent. in their ability to lift weights; not by exercise but simply by the improvement of the quality of the muscles. You are likely to find that you may be able to lift easily and without resultant soreness twice the amount you have been able to lift while full of poisons. This is capable of easy demonstration, as has been shown by Mr. Stapleton, formerly of Yale University gymnasium, who enlisted for the experiments under Professor Chittenden at Yale, five years ago, he went into them half-heartedly, because he felt himself to be already in superior physical condition. But he soon found that his energy, strength, and endurance were greatly increased. This improvement, which amounted to a full 100 per cent. or more, came, not as the result of more training, but as the result of the improvement of the quality of the muscles effected by keeping poisons out of the body.

Mr. Granger, of the Battle Creek Sanitarium, as the result of only thirty days of following the right method of eating—and he was in good condition before he started it—was able to perform deep-knee bending (dropping the body down to the heels and lifting it to full height) five thousand and two times consecutively, which required two hours and nineteen minutes of unceasing dropping and lifting. He had never been able to go beyond three thousand dips previously, and on former occasions he had stopped because he could not go on. After thirty days of taking food in the manner recommended he was able to go five thousand and two times, and then he stopped because everybody detailed to watch him grew tired—as he expressed it,

"I felt sorry for those people who where watching me." Then he ran down stairs and plunged into a swimming tank and felt no soreness whatever afterward.

I myself, as a matter of demonstration, am frequently asked to "make good." Arriving in this country some time ago, after having spent fifteen months in India and in the tropics around the equator, and after experiencing all sorts of adventures, without any systematic training at all, I was called upon to try a new endurance machine at the Yale gymnasium, the invention of Professor Irving Fisher. I went through the test, not realizing that I had accomplished anything remarkable, but I found that I had broken all previous records for this test. It was a fair test on my part because I was not expecting to break records, and I assure you that I felt no inconvenience after coming off the platform. I felt a little lighter than usual, and the next day expected to feel soreness, but did not. I simply give this as an example of a test for muscular endurance. It has been demonstrated in many instances that this increased muscular capacity is purely the result of dietetic regulation, and it is within the power of everyone to make these experiments for themselves.

Fatigue manifests itself under ordinary conditions where there are poisons in the circulation and hunger manifests itself by watering of the mouth. For instance, if you have been busy at work and have developed a keen appetite so that the fumes of corn bread being taken out of the oven will make you whinny like a horse, you may be sure you have a proper, a healthy appetite.

Many people think that the joy of eating consists in the act itself. Some say they do not want to adopt my system, if they have to give up all the pleasure of eating, but I answer that until they do so they will not know the full pleasure of eating! The chief enjoyment in regard to any pleasure is complete satisfaction—the satisfaction of having had all you want. Satisfaction is the acme of pleasure. You may enjoy the anticipation of a meal, and you may think that it is, like a good thirst to a toper, worth a great deal. When the time for eating comes you may enjoy the taste of your food greatly, but the greatest comfort and keenest pleasure is when you have satisfied the appetite and do not want any more. In other words, the acme of all enjoyment is not wanting. Hence, if you can arrange your eating so as to enjoy taste twice a day for thirty minutes, you can have full enjoyment of the maximum pleasure of complete satisfaction for the rest of the day; you have gained decidedly in economy.

Many have thought and declared that they never could spare the time that proper eating of food requires. In this they are very much mistaken. During the time I was under test at Yale, taking the exercises of the 'varsity crew, and engaging in a great many other activities, it took me only from twenty-four to twenty-eight minutes a day to eat my food, and I was nourished fully, losing no weight, but on the contrary gaining slightly. Of course, while I was taking my sustenance I was not talking, but was attending strictly to business. The twenty-four to twenty-eight minutes a day were divided into two meals of twelve to fourteen minutes each, one at noon and the other at six in the evening, and I easily maintained full nutrition during this strenuous test. When a person becomes accustomed to using his digestive faculties he can nourish himself readily within thirty to forty minutes a day, and consequently there is no question of waste of time.

I am not a guide for anybody, but I will tell you what I ate to-day. I took my first meal at 10.30 A. M.—after having worked with the utmost strenuousness from half-past three in the morning—and that meal consisted of a small piece of bluefish, hashed brown potatoes, two cups of milk, a little coffee in the milk and a great deal of sugar, say three or four lumps to each cup, a warm corn-bread muffin and butter. That is the only food I have taken to-day, and I think it will be quite late in the evening before I shall want my second meal. This may seem simplicity itself, but I do not know anybody who is occupied more strenuously than I am at the present time. This propaganda that I have become engaged in has brought upon me an enormous strain of work. It is the sort of work that no one can do for me, and beginning work as early as I do, I have to do all my own writing. Some years ago I wrote all of my manuscript by machine; paralysis in one hand compelled me to use a typewriter. Frequently I could use only one hand. I can assure you that it was a distressful condition of incapacity. Now I have learned to use the pen double-handed. The last time I went to Europe, in the fourteen days that I was on the trip I wrote seventy thousand words by hand.

I have practically abandoned meat, because I like other things better. I discontinued habitual meat-eating in the first place because I ceased to want it, and in the second place because I do not believe it to be good for me—at least I seem better without it. Consequently, while I am not quite a professed vegetarian, I must say that all the arguments I know are in favor of the vegetarian diet.

I drink whenever I am thirsty. If I have thirst at the time I am eating I drink to quench the thirst, and have never had any bad results.

I will tell you the plan we follow in our home in Venice. There is only one set meal, and that is served anywhere from twelve to one o'clock, because that happens to be the most convenient time for everybody to sit down and enjoy food. I will tell you also something with regard to a little grandchild, who is something of a curiosity as he was born since this system came into operation, and is being carefully observed. He is now five years old. He was nursed for the full term and only with the eruption of the teeth was he weaned. From that time on he has always chosen his own food. He is provided with a sufficient range of food to choose from, but he has limited it to two or three things. One is a sort of food that he calls "brown egg," material that is rich in proteid, and the other is rice pudding, composed chiefly of starchy ingredients. He is asked an hour or so before his usual time to have his food, "What do you want to-day?" and he will answer, "I want my rice pudding." In that case it is evident that he has all the proteid supply he needs. Another time he will ask for his "brown egg," and at other times he will demand both. He is allowed to choose without suggestion of choice whatever. He is the boy with whom Sir Michael Foster was acquainted and about whom Sir Michael siad: "If the child is born at all and is not a monstrosity, the physiology of nutrition will have to be re-written." The child was born, and he is not a monstrosity. When three years of age he ate three times a day. Then, however, he showed a strong preference for play in the morning rather than for eating. The six months following he was in the habit of taking no food until twelve o'clock. Then he had his "rice pudding" or "brown egg," and nothing else until five o'clock, when he took his second and last meal of the day. As I have said, we have one regular meal at twelve or one o'clock, but there is always something prepared, and if anyone is hungry he may go to the cupboard and find something agreeable. That simplifies the question of household economics so much that our cook protested, because he feared losing his art entirely, and declared that he would not work where he did not get more practice.

